# Retrospective Evaluation of Acute Kidney Injury After Zoledronic Acid Administration to Dogs With Malignant Osteolysis

**DOI:** 10.3389/fvets.2021.647846

**Published:** 2021-07-02

**Authors:** Sarah A. Vidal, Katherine A. Skorupski, Jennifer L. Willcox, Carrie A. Palm, Jenna H. Burton

**Affiliations:** ^1^William R. Pritchard Veterinary Medical Teaching Hospital, School of Veterinary Medicine, University of California, Davis, Davis, CA, United States; ^2^Department of Surgical and Radiological Sciences, School of Veterinary Medicine, University of California, Davis, Davis, CA, United States; ^3^Department of Medicine and Epidemiology, School of Veterinary Medicine, University of California, Davis, Davis, CA, United States

**Keywords:** aminobisphosphonate, azotemia, canine, pain management, zoledronate

## Abstract

Zoledronic acid (ZOL) is an intravenous bisphosphonate indicated for the use of hypercalcemia of malignancy and management of bony metastases. Its therapeutic effect lies in the targeting of malignant osteoclasts; however, administration can be associated with renal toxicity. The objective of this retrospective study was to evaluate the frequency and severity of acute kidney injury (AKI) following ZOL administration in a cohort of cancer-bearing dogs. A pharmacy search was conducted to identify dogs that received a dose of ZOL between June 2016 and July 2019. Inclusion criteria included baseline and post-treatment chemistry panels. Medical records were reviewed to obtain clinical data including signalment, dose, dosage, number of treatments administered, and changes in renal function. Forty-four dogs met the inclusion criteria. Median number of doses administered was three [interquartile range (IQR), 2–5]. The median highest creatinine value occurred after a median of one dose (IQR, 1–2 doses) compared with the median highest value of blood urea nitrogen, phosphorus, and potassium, which occurred after a median of two doses (IQR, 1–3). Six (13.6%) dogs developed an AKI, and one dog (2.3%) had progression of an existing azotemia after treatment with ZOL was initiated. Two dogs (4.5%) had ZOL treatment discontinued secondary to development of azotemia. Use of concurrent administration of non-steroidal anti-inflammatory drugs or anesthesia did not significantly increase the risk of AKI in this cohort of dogs. Acute kidney injury is observed infrequently in cancer-bearing dogs treated with ZOL and is generally mild to moderate in severity; discontinuation of ZOL due to AKI is uncommon.

## Introduction

Bisphosphonates are synthetic analogs of naturally occurring inorganic pyrophosphate compounds whose therapeutic effect inhibits osteoclasts, reducing pathologic bone resorption without inhibiting bone mineralization (1). Following administration, bisphosphonates bind to hydroxyapatite particles in bone and are subsequently released and endocytosed during osteoclastic-mediated resorption, resulting in apoptosis of osteoclasts and inhibition of bone resorption (1, 2). Bisphosphonates are currently widely used in the treatment of osteoporosis, Paget's disease of bone, hypercalcemia of malignancy, and reduction of skeletal events in the management of bony metastases in people (3–8).

In veterinary medicine, bisphosphonates are most commonly used in the management of humoral hypercalcemia of malignancy (HHM) and pain secondary to malignant osteolysis. The second-generation bisphosphonate, pamidronate, has been widely used both as a single agent or in conjunction with chemotherapy or radiation therapy for pain management of bone tumors in dogs (9–13). Zoledronic acid (ZOL) is a third-generation, intravenous bisphosphonate, and the addition of nitrogen to its molecular structure provides ZOL increased anti-resorptive potency compared with earlier generations of bisphosphonates (14). It also has the ease of being administered intravenously over 15 min compared with earlier intravenous (IV) bisphosphonates, such as pamidronate, which is generally administered IV over 2 to 4 h, and over 24 h in some human patients (15). FDA approved in 2001, ZOL was initially cost prohibitive for broad use in veterinary medicine until recent years. Investigations regarding the efficacy of ZOL in managing bone pain and HHM in dogs are currently limited, but preliminary investigations suggest clinical benefit for managing both these conditions (16, 17).

Commonly reported adverse events secondary to ZOL administration in people include acute systemic inflammatory reactions and bone pain. Acute and chronic renal failure are also reported side effects of ZOL therapy (18, 19). Bisphosphonates are excreted unchanged through the kidney in a concentration- and dose-dependent manner, and rapid excretion of the drug exposes the kidney to high concentrations of bisphosphonates, which may result in renal injury (19). Results of phase III clinical trials of ZOL demonstrate that 9 to 15% of people treated with ZOL had evidence of renal deterioration as characterized by elevations in serum creatinine (3). Renal toxicity after a single dose of ZOL occurs, on average, 11 days after treatment (20). As risk of renal toxicity increased with higher doses and shorter infusion times in people, recommended ZOL dosing for people with multiple myeloma and solid tumors is 4 mg IV over no <15 min every 3 to 4 weeks for patients with normal kidney function (18). It is also recommended that blood work be used to monitor metabolic parameters including creatinine, phosphorus, and calcium following initiation of therapy (18).

Clinical impression suggests that ZOL administration to dogs appears to be well-tolerated; however, adverse events secondary to ZOL administration to dogs have not been widely investigated. In a 2008 study by Fan et al., nine dogs with osteosarcoma treated with ZOL did not have statistically significant increases in blood urea nitrogen (BUN), creatinine, calcium, phosphorous, or potassium after ≥2 doses of ZOL (16). As our institution has shifted to primarily using ZOL due to comparable cost and increased ease of administration compared with pamidronate, the objective of this retrospective study was to evaluate the frequency and severity of acute kidney injury following administration of ZOL to dogs for management of malignant osteolysis.

## Materials and Methods

A pharmacy search was performed at the University of California, Davis Veterinary Medical Teaching Hospital (UCD VMTH), to identify dogs prescribed with ZOL between June 2016 and July 2019. Medical records were reviewed, and cancer-bearing dogs with primary and metastatic bone lesions were included in the study if there was a pre-treatment chemistry panel prior to treatment and at least one follow-up chemistry panel performed after ZOL administration. Cases were excluded from the study if dogs received ZOL for management of hypercalcemia or if pre- and post-ZOL chemistry panels were not available for review. Dogs that had previously received pamidronate prior to ZOL treatment were included as long as other inclusion criteria were met.

Information abstracted from the medical record included patient demographics and diagnosis as well as ZOL dose and dosage per treatment, dates of pre-treatment bloodwork and first treatment, number of treatments, and frequency of zoledronate administration. To assess renal parameters over the course of treatment, baseline pre-treatment values for blood urea nitrogen (BUN), creatinine, total calcium, potassium, and phosphorus were recorded. All chemistry panels performed after initiation of ZOL treatment were reviewed and the highest BUN, creatinine, potassium, phosphorus, as well as the highest and lowest calcium throughout the course of ZOL treatment were recorded along with the date that these elevations occurred. Concurrent administration of non-steroidal anti-inflammatory drugs (NSAID), previous pamidronate administration, and concurrent anesthetic events were also documented. Increases in creatinine were graded according to Veterinary Cooperative Oncology Group—Common Terminology Criteria for Adverse Events (VCOG-CTCAE) Metabolic/Laboratory category. VCOG-CTCAE Renal/Genitourinary category and the International Renal Interest Society (IRIS) Acute Kidney Injury (AKI) grading criteria were used to assess severity of renal injury; both of these grading schemes are based on increases in creatinine which may or may not be outside the normal reference range (21, 22). A sustained increase in creatinine ≥0.3 mg/dl from baseline or increase in creatinine >1.6 mg/dl following ZOL administration was considered to indicate evidence of kidney injury as based on these guidelines. If ZOL treatment was discontinued in any case, the reason for discontinuation was recorded.

Continuous data were assessed using Shapiro–Wilk test for normality and reported as mean and standard deviation (SD) for normally distributed data or median and interquartile range (IQR) for non-normal data. Categorical data were described using frequencies and percentages. Difference between baseline and follow-up blood work values were assessed using Wilcoxon matched pairs signed rank test. Differences among groups with continuous data were assessed using a Mann–Whitney test. Fisher's exact test was used to assess differences for variables with two categories. To assess the effect of additional risk factors that may have contributed to AKI development (anesthesia, NSAIDs, or previous pamidronate), dogs were categorized based on the presence of additional risk factors for AKI. Dogs that only had ZOL administration were placed in category 0, and dogs that had contemporaneous anesthesia, NSAIDs, and previous pamidronate were placed in category 3. Dogs in category 1 had one of the three additional risk factors for AKI, and those in category 2 had any combination of two additional risk factors. Due to the small sample size and numbers of dogs in categories 0 and 3, the categories were combined in 0–1 and 2–3 additional risk factors for AKI. *p*-values < 0.05 were considered statistically significant. All analyses were performed with the aid of a statistical software, GraphPad Prism 8.4.1 for macOS (La Jolla, CA, USA).

## Results

One hundred twenty-seven dogs were identified by pharmacy search as having received at least one dose of ZOL between June 2016 and July 2019. Of these, 83 were excluded from analysis; 17 received ZOL due to hypercalcemia, and 66 dogs did not meet necessary blood work requirements. In total, 44 dogs were eligible for inclusion having received at least one dose of ZOL for pain management with baseline and follow-up chemistry panel. All blood work was performed at UCD VMTH with the exceptions of eight dogs (18%) that had baseline chemistry panels performed at their primary care veterinarian.

The mean age of the patient population was 9.76 years old (SD ± 2.28 years), and the median weight was 34.7 kg (IQR, 21.2–50.9 kg). There were 16 (36.3%) spayed females, 26 (59%) neutered males, and two (4.5%) intact males. Breeds included mixed (*n* = 8, 18%), golden retriever (*n* = 5, 11%), Saint Bernard (*n* = 4, 9.1%), rottweiler (*n* = 3, 6.8%) and two dogs (4.5%) of each of the following breeds: vizsla, Labrador retriever, German shepherd, Great Dane, and dachshund. The remaining dogs were represented by other breeds (*n* = 14, 32%). Primary bony tumors comprised the majority of tumors (*n* = 31, 70.5%) followed by metastatic urogenital tumors (*n* = 4, 9.1%), multiple myeloma (*n* = 2, 4.5%), nasal carcinomas (*n* = 2, 4.5%), and one dog of each (*n* = 5, 11.4%) of the following tumors with bony invasion or metastasis: synovial myxoma, squamous cell carcinoma of the ear canal, soft tissue sarcoma, subcutaneous carcinoma, and apocrine gland anal sac adenocarcinoma (AGASACA).

The median number of doses of ZOL administered was three (IQR, 2–5), with one dog receiving 24 ZOL treatments in total. The median ZOL dosage was 0.1 mg/kg (IQR, 0.09–0.1), and the median ZOL dose was 3.52 mg (IQR 2.1–4.0). Fourteen dogs (31.8%) had a calculated dose that exceeded 4 mg, but the total dose administered was capped at 4 mg per clinician preference. The ZOL dose exceeded 4 mg for five dogs (11.3%); all five were treated with a dose of 8 mg, and the mean dosage for these five dogs was 0.11 kg/kg (SD ± 0.03). The median treatment interval for the 39 dogs (88.6%) receiving more than one dose was 21.4 days (IQR, 17–24 days). Three dogs (6.8%) were previously treated with pamidronate; these dogs received a total of 1, 2, and 11 doses of pamidronate prior to treatment with ZOL. Concurrent NSAIDs were administered to 34 dogs (77.2%). The date of NSAID initiation was unknown in 4 (11.8%) of the 34 dogs receiving concurrent NSAID. With the remaining 30 dogs (88.2%), NSAIDs were started prior to therapy in 23 dogs (67.6%), on the day of first ZOL in 4 dogs (11.8%), and during the course of ZOL in the remaining 3 dogs (8.8%). NSAIDs were initiated a median of 31.0 days prior to ZOL (IQR, 20–46 days). Contemporaneous anesthetic events were also common with 29 dogs (65.9%) having undergone general anesthesia during the course of ZOL administration or in the timeframe between when baseline blood work was obtained and the first administration of ZOL. Of dogs undergoing anesthetic events, 26 (89.7%) were anesthetized for radiation therapy with 20 dogs (70.0%) receiving radiation to the appendicular skeleton, five (17.2%) to the axial skeleton, and one (3.4%) to the prostate. The remaining three dogs (10.3%) underwent anesthesia for computed tomography.

Median baseline and highest values for renal parameters are reported in Table 1. Baseline chemistry panels were performed a median of 1 day (IQR, 0–15 days) prior to ZOL administration. A baseline urine specific gravity (USG) was available in 19 dogs (43.2%) with a mean of 1.029 (SD ± 0.012). Baseline symmetric dimethylargine (SMDA) testing was not performed for any of the dogs. Following ZOL treatment, creatinine and BUN were statistically increased from baseline (*p*-value 0.002 and 0.0002, respectively) when evaluating all 44 dogs despite remaining within the normal reference range; no statistical differences between baseline and highest values of calcium, potassium, and phosphorus were observed. The median highest value of creatinine was 1.1 mg/dl, which occurred after a median of one dose (IQR, 1–2) compared with the median highest value for BUN at 25.9 mg/dl, which occurred after a median of two doses (IQR, 1–3).

**Table 1:** Baseline and median highest renal and electrolyte values for dogs following zoledronic acid administration.

**Parameter**	**Reference interval**	**Median baseline value (IQR)**	**Median highest value (IQR)**	***p*-Value**	**Median # of days after treatment initiation (IQR)**	**Median # of doses (IQR)**
Creatinine (mg/dl; *n* = 44)	0.8–1.5	1.0 (0.8–1.2)	1.1 (0.9–1.3)	0.002	36 (22–61)	1 (1–2)
BUN (mg/dl; *n* = 44)	11–33	17 (13–22.8)	21.0 (15–28)	<0.001	54 (27–71)	2 (1–3)
Total calcium (mg/dl; *n* = 43)	9.6–11.2	10.0 (9.7–10.4)	10.3 (9.8–10.8)	0.065	55 (24–75)	2 (1–2)
Phosphorus (mg/dl; *n* = 42)	2.6–5.2	4.3 (3.8–4.8)	4.0 (3.5–4.7)	0.446	55 (24–90)	2 (1–3)
Potassium (mmol/L; *n* = 39)	3.6–4.8	4.6 (4.4–5.0)	4.8 (4.5–5.2)	0.238	56 (27–83)	2 (1–3)

*BUN, blood urea nitrogen; IQR, interquartile range*.

Treatment with ZOL was initiated in two dogs (4.5%) with creatinine values above the upper limit of normal prior to treatment; both of these dogs had advanced urogenital tumors with bony metastasis and only received a single dose of ZOL. Both had an elevated baseline creatinine of 1.8 mg/dl. As no baseline urinalysis or SDMA was performed, underlying chronic kidney disease could not be ruled out for either dog. Creatinine decreased to within normal limits 19 days following ZOL treatment for one dog; the other dog had progression of AKI with creatinine increased to 2.3 mg/dl 16 days after ZOL administration.

In addition to the dog with progression of preexisting azotemia during the course of the study, six non-azotemic dogs (13.6%) had a sustained increased in creatinine of ≥0.3 mg/dl from baseline, consistent with AKI based on the IRIS grading scheme. Increases in creatinine were also graded using the VCOG-CTCAE Metabolic/Laboratory category (creatinine) and Renal/Genitourinary category (AKI), and due the inconsistencies in grade determination using the VCOG criteria (Table 2), the IRIS AKI grading system is used for the remainder of the manuscript to describe the severity of AKI. For these six dogs, the median baseline creatinine was 0.85 mg/dl (IQR, 0.4–1.2 mg/dl), and the median highest value of creatinine was 1.7 (IQR, 1.1–1.9 mg/dl) mg/dl. Of the seven total dogs to develop an AKI (six non-azotemic and one azotemic at baseline), three dogs (6.8%) had a grade I AKI where creatinine increased ≥0.3 mg/dl from baseline but remained <1.6 mg/dl, and four dogs (9.1%) had a grade II AKI (creatinine 1.7–2.5 mg/dl), based on the IRIS grading scheme. These increases in creatinine were observed after a median of one dose of ZOL (IQR, 1–2 doses), and a mean of 35 (SD ± 30) days after treatment initiation. The median number of total doses received was two (IQR, 2.0–4.0 doses) for dogs that developed an AKI.

**Table 2:** Variability in assessment of renal toxicity in seven dogs after initiation of zoledronic acid treatment using the International Renal Interest Society (IRIS) Acute Kidney Injury (AKI) and Veterinary Cooperative Oncology Group-Common Terminology Criteria for Adverse Events (VCOG-CTCAE) grading systems and assessment of likeliness of AKI to zoledronic acid administration.

**Patient #**	**Baseline creatinine (mg/dl)**	**Highest creatinine (mg/dl)**	**IRIS AKI grade**	**VCOG AKI grade**	**VCOG creatinine grade**	**Attribution to ZOL**
1	0.5	1	1	1	2	Unlikely
2	0.4	1.1	1	2	2	Possible
3	0.4	1.6	1	3	3	Unlikely
4	1.3	1.8	2	1	1	Possible
5	1.2	1.9	2	1	1	Likely
6	1.2	1.9	2	1	1	Likely
7	1.8	2.3	2	1	2	Possible

*AKI, acute kidney injury; IRIS, International Renal Interest Society; VCOG-CTCAE, Veterinary Comparative Oncology Group-Common Terminology Criteria for Adverse Events; ZOL, zoledronic acid*.

None of the dogs were clinical for AKI and diagnostic tests to determine the etiology of renal injury were inconsistently performed for dogs that developed an AKI while treated with ZOL. A grade I AKI was suspected to be pre-renal in nature for one dog that presented for acute paraparesis and was determined to be 5–7% dehydrated at the time the AKI was documented, and another dog was presumed to have developed a post-renal grade II AKI secondary to severe peritonitis and retroperitonitis 11 days post-laparotomy with resection and anastomosis to treat a duodenal perforation and septic peritonitis. Two dogs (4.5%) with grade II AKI had ZOL discontinued due to concerns of renal injury secondary to ZOL; both of these dogs were receiving concurrent NSAIDs and had been anesthetized on the same day or 1 day after the first ZOL administration. Of the remaining three dogs, one had a grade I AKI that developed and persisted over the course of 24 treatments of ZOL. Another dog developed a grade II AKI after two doses of ZOL and continued to receive a total of four doses despite persistence of the grade II AKI. Treatment with ZOL was discontinued after amputation of the affected limb and the creatinine returned to baseline value 3 months after the last ZOL treatment. The final dog, which had an extensive bladder tumor with pulmonary and suspected bony metastasis, previous placement of bilateral ureteral stents, history of recurrent urinary tract infections, and pre-existing azotemia at the time of ZOL administration, developed a grade II AKI 16 days after a single dose of ZOL. Because of the dog's history of urinary tract infections, a urine culture was performed when the AKI was noted, and no growth was observed. The dog had minimal clinical benefit from the ZOL, so no additional doses were administered, and the dog was lost to follow-up after AKI diagnosis. Possible attribution of ZOL for each of these dogs is provided in Table 2.

To assess potential differences between the dogs that developed an AKI (*n* = 6) and those that did not (*n* = 37), the following parameters were evaluated: ZOL dosage, ZOL dose, baseline creatinine, baseline BUN, administration of concurrent NSAIDs, contemporaneous anesthetic episodes, and previous pamidronate administration. The dog with a pre-existing azotemia that persisted after ZOL treatment was not included in this analysis as pre-existing renal impairment increases the risk of development ZOL-induced AKI in people (18). There was no significant difference between the two patient populations on any of these parameters (Table 3). The effect of additional risk factors such as contemporaneous anesthesia, concurrent NSAID administration, or previous pamidronate were assessed, and dogs were categorized based on their number of additional risk factors for AKI at the time of ZOL treatment. Five (11.6%) of the 43 dogs had no additional risk factors for AKI development (category 0), 12 (27.9%) dogs had one risk factor (three contemporaneous anesthesia, nine concurrent NSAID), 24 (55.8%) had two risk factors (*n* = 23 anesthesia and NSAID therapy, n = 1 anesthesia and pamidronate), and two (4.7%) dogs received concurrent NSAIDs and anesthesia, and were previously treated with pamidronate (category 3). Due to the small numbers of dogs in categories 0 and 3, the categories were combined in 0–1 and 2–3 additional risk factors for AKI. There was no significant difference between the odds ratio for AKI occurrence between category 0–1 and 2–3 [*p* = 0.419, OR 2.19 (95% CI 0.52–9.53)].

**Table 3:** Comparison of various parameters between 37 dogs without renal toxicity and 6 dogs non-azotemic at baseline with documented AKI following zoledronic acid administration.

**Parameter**	**Group**	**Median (IQR) or frequency (%)**	***p*-value**	**95% CI or OR (95% CI)**
**Dosage (mg/kg)**	No toxicity	0.1 (0.09–0.11)	0.363	−0.02 to 0.01
	AKI	0.1 (0.08–0.10)		
**Dose (mg)**	No toxicity	3.8 (2.4–4.0)	0.387	−2.1 to 0.67
	AKI	2.7 (1.8–4.0)		
**Number of doses**	No toxicity	3.0 (2.0–5.5)	0.348	−3.0 to 1.0
	AKI	2.5 (1.0–9.0)		
**Baseline creatinine (mg/dl)**	No toxicity	1.0 (0.8–1.2)	0.494	−0.6 to 0.3
	AKI	0.85 (0.4–1.2)		
**Baseline BUN (mg/dl)**	No toxicity	17.0 (13.0–22.0)	0.871	−8.0 to 8.0
	AKI	18.0 (9.5–26.3)		
**Concurrent NSAID**	No toxicity	30 (81.1%)	0.589	0.467 (0.08 to 2.89)
	AKI	4 (66.7%)		
**Contemporaneous anesthetic episode(s)**	No toxicity	25 (67.6%)	>0.999	0.960 (0.193 to 5.60)
	AKI	4 (66.7%)		
**Previous pamidronate**	No toxicity	3 (8.1%)	>0.999	0.00 (0.00 to 7.52)
	AKI	0		
**Risk factors for AKI**	0–1	17.6% had AKI	0.667	1.64 (0.34 to 7.72)
	2–3	11.5% had AKI		

*AKI, acute kidney injury, BUN, blood urea nitrogen; CI, confidence interval; IQR, interquartile range; OR, odds ratio; NSAID, non-steroidal anti-inflammatory drugs*.

## Discussion

This retrospective study aimed to determine the frequency and severity of AKI observed in dogs undergoing ZOL treatment for management of malignant osteolysis. Using IRIS criteria, development of AKI was documented in seven (15.9%) dogs administered with at least one dose of ZOL, with ZOL administration discontinued in two dogs (4.5%) because of suspected ZOL-induced kidney injury. Underlying risk factors identified for people treated with ZOL include advanced cancer, previous bisphosphonate administration, and use of NSAIDs (20). Concurrent risk factors for the development of AKI such as NSAIDs, concurrent anesthesia, and previous pamidronate administration did not increase the risk of AKI in this population of dogs though risk of Type 2 error cannot be excluded in this study.

One of the mechanisms of action of kidney damage following ZOL is acute tubular necrosis following excretion of unchanged drug by the kidneys (23). Phase III clinical trials in people established renal toxicity secondary to ZOL administration to be dose- and time-dependent with patients that received 8 mg of ZOL or received the drug as a more rapid 5-min infusion more likely to develop renal toxicity. These findings resulted in the label prescribing dosage information of 4 mg as an IV infusion over no less than 15 min (4, 5, 18). This dose-dependent toxicity was also generally observed in rats, where large doses of bisphosphonates resulted in exposure of the kidneys to large concentrations of drug and subsequent dose-dependent and time-dependent increase in BUN and creatinine (24). At our institution, administration of ZOL to dogs with an IV infusion over 15 min is similar to ZOL administration to people, though details of ZOL administration in the electronic medical records were not available for all dogs. Reported ZOL dosage for dogs has ranged from 0.1 to 0.25 mg/kg in previous published studies, and the dosages administered in this study were more consistent with the lower end of that range (16, 17), and there was no association with ZOL dose or dosage identified for this patient population.

The IRIS AKI grading system was initially developed in 2013 and revised in 2016 to assess and report renal injury and subsequent response to therapy in a systematic manner. We elected to focus on this grading scheme in reporting AKI due to the variability in grading when using the VCOG-CTCAE. Using the IRIS AKI grading, a grade I AKI is defined as a non-azotemic dog with a <1.6 mg/dl creatinine, but creatinine increase ≥0.3 mg/dl from baseline (22). Use of IRIS AKI scheme allowed for reporting of mild creatinine increases in non-azotemic dogs and ensured that AKI development was not underestimated for dogs that had creatinine increases within the normal reference range.

Kidney injury, as characterized by a 0.5 mg/dl increase from normal baseline creatinine, or a 1.0 mg/dl increase in an abnormal creatinine, occurs in 9 to 15% of people who received 4 mg of ZOL over 15 min as treatment for bony metastases (18, 20). In our study, 15.9% of dogs were documented to develop an AKI using the IRIS grading system, and likeliness of association with ZOL administration was assessed to be “unlikely” for two dogs, “possible” for three dogs, and “likely” for two dogs. While known risk factors for AKI, such as bisphosphonate administration and NSAID use, for people treated with ZOL were not found to be statistically significant in this study, Type 2 error cannot be excluded. AKI development is potentially multifactorial in this cohort of dogs and larger studies are needed to identify risk factors for AKI development in dogs treated with ZOL.

Two (28.6%) of the seven dogs with an AKI went on to receive additional doses of ZOL following AKI development without further increase in AKI grade. Additionally, treatment with ZOL was initiated in two dogs despite the presence of an elevated creatinine at the time treatment was initiated. Dose reductions are recommended for people with reduced renal function prior to initiation of ZOL treatment, and likewise, it is recommended that people who develop serum creatinine elevations during ZOL treatment have therapy delayed until creatinine returns to within 10% of baseline (18). Neither dose reductions nor treatment delays were performed for any dogs in this study, with the exception of the two dogs where ZOL treatment was discontinued due to suspected renal toxicity secondary to ZOL. Overall, the majority of dogs in this study did not have clinically significant changes to creatinine after ZOL administration, with median values of these parameters remaining within reference range. There were no hospitalizations secondary to renal value increases in this cohort of dogs. Ultimately, the documented AKIs did not alter the course of palliative treatment in majority of dogs with advanced neoplasia and metastatic disease in this study, and therefore, associated kidney injury was not considered to be clinically significant in this patient cohort.

Additional limitations of this study include those inherent to retrospective studies, including a small sample size, inconsistent and missing information within the electronic medical record, and variable timing between ZOL treatments and follow-up blood work. As extent and type of diagnostics performed were limited at baseline and even after documented of an AKI, it is possible that without consistent baseline urinalyses and SDMA testing, early pre-existing renal disease may have been missed in this cohort of dogs prior to starting treatment with ZOL. Hydration status was inconsistently noted in the records, and while clinical practice is to administer ZOL to euhydrated dogs, it is possible that creatinine changes may have been overestimated in the grading of AKI in the face of concurrent dehydration. It is also possible that an AKI attributable to ZOL may have been overestimated with the lack of additional diagnostics. In such an instance, urinary tract ultrasound imaging or urine culture may have indicated a secondary cause for renal value changes attributable to progression of disease or pyelonephritis instead of ZOL administration. Consistent evaluation of urinalyses, the inclusion of SMDA testing, along with routine monitoring of renal panel may provide a more accurate assessment of the nephrotoxic potential of ZOL in dogs for future studies. Finally, as body muscle scores were not recorded, it is possible that loss of muscle mass could have led to less severe increases in creatinine over time and, therefore, an underestimation of kidney injury.

In summary, results of this study demonstrate that acute kidney injury is observed infrequently in cancer-bearing dogs treated with ZOL, with only 4.5% of this population having ZOL treatment discontinued for suspected renal toxicity. As dogs with osseous neoplasia often receive concomitant treatment with NSAIDs and palliative radiation, regular assessment of renal function is advisable throughout ZOL treatment to monitor for development of AKI.

## Data Availability Statement

The raw data supporting the conclusions of this article will be made available upon request.

## Ethics Statement

Ethical review and approval was not required for the animal study because this study was a retrospective review of veterinary medical records. Written informed consent for participation was not obtained from the owners because this study was a retrospective review of veterinary medical records.

## Author Contributions

The study concept was developed by JB, KS, and JW. Data acquisition, analysis, and drafting of the manuscript were performed by SV and JB. The manuscript was reviewed and edited by CP, JW, and KS. All authors contributed to the article and approved the submitted version.

## Contributions to Science

Zoledronic acid is increasingly used in veterinary oncology to manage hypercalcemia of malignancy and pain secondary to malignant osteolysis, however the adverse event profile in dogs has not been thoroughly investigated at this time. Zoledronic acid is excreted unchanged through the kidneys and development of renal toxicity is a significant side effect in people receiving the drug. Clinical impression suggests that zoledronic acid is well tolerated in veterinary patients, but as its use is increasing by veterinary oncologists to manage hypercalcemia of malignancy and pain secondary to malignant osteolysis in their canine patients, we sought to evaluate the risk of renal toxicity in dogs receiving the drug for pain management of bone pain secondary to cancer. This study concludes that acute kidney injury is uncommon in dogs with cancer but supports regular monitoring of kidney function in dogs receiving this drug.

## Conflict of Interest

The authors declare that the research was conducted in the absence of any commercial or financial relationships that could be construed as a potential conflict of interest.
